# Supporting Mental Health and Physical Wellbeing Among Nursing Students Through Yoga: A Mixed-Methods Study

**DOI:** 10.3390/nursrep15080305

**Published:** 2025-08-20

**Authors:** Beverley Martin, Blake Peck, Liz Ryan, Andy Davies, Daniel Terry

**Affiliations:** 1 School of Nursing and Midwifery, University of Southern Queensland, Ipswich, QLD 4305, Australia; b.peck@federation.edu.au (B.P.); liz.ryan@unisq.edu.au (L.R.); daniel.terry@unisq.edu.au (D.T.); 2 Institute of Health and Wellbeing, Federation University Australia, Ballarat, VIC 3350, Australia; 3 Centre for Health Research, University of Southern Queensland, Springfield, QLD 4300, Australia

**Keywords:** yoga, stress, anxiety, depression, holistic, self-care, students, nurse

## Abstract

**Background/Objectives**: The mental health and engagement of nursing students are critical for academic success and professional readiness. This study explored the impact of an 8-week Hatha yoga programme on undergraduate nursing students’ engagement, depression, anxiety, stress, procrastination, sense of belonging, and intention to drop out. **Methods**: A mixed-methods design was employed, collecting data pre- and post-intervention between July 2023 and November 2024. Fifty-nine students initially enrolled in the study, with fourteen completing the full yoga programme and post-intervention assessments. Quantitative data were analysed using descriptive and non-parametric statistical tests, while qualitative data from semi-structured interviews were analysed thematically to capture students’ lived experiences. The study has been guided by the STROBE guidelines. **Results**: Statistically significant reductions in depression, anxiety, and stress were observed among students who completed the yoga programme. Improvements in behavioural and emotional engagement were also noted. Qualitative findings revealed that yoga fostered a renewed sense of wellbeing, self-care, and resilience, particularly among students balancing academic, work, and family responsibilities. **Conclusions**: Participation in an 8-week yoga programme was associated with reduced psychological distress and enhanced engagement among nursing students. These findings support the integration of holistic self-care practices into nursing curricula to promote student wellbeing and academic persistence.

## 1. Introduction

Examining curriculum for Baccalaureate nursing programmes has highlighted a gap in learning pedagogy, which encompass strategies for developing non-cognitive and self-care skills [1]. The nursing profession intrinsically cares for people, which is personally demanding, but should not be at the cost of nurses’ wellbeing [2]. The importance of teaching students’ holistic approaches to manage and prevent physical and emotional exhaustion cannot be overestimated [2]. Such endeavours are of interest to educators given the increasing awareness of mental health issues among students and highlight the need to build self-care strategies into the curriculum of nursing programmes. These strategies enable nursing students to stay mentally and physically fit, which includes demonstrated benefits and activities of self-care as they enter the workforce [2,3].

In addition to preparing nursing students for the workforce, dropout rates amongst nursing students in Baccalaureate programmes continue to rise globally, and novel approaches are required to better support undergraduate student success [4]. One such approach emerging is building into nursing curricula non-academic factors, such as self-care skills, to assist students as they traverse their studies and enable them to better manage and cope as they enter the workforce. The elements of self-care can be developed as students academically learn, through skills such as improved self-awareness, self-regulation, study techniques, work habits, time management, seeking help, and problem-solving abilities [5]. Developing these skills may safeguard the non-cognitive factors of emotional, mental, and physical wellbeing of the individual [6]. Non-cognitive factors include a broad range of personality, motivation and other psychological elements, which include traits such as perseverance, self-control, social skills and emotional regulation, and crucial for personal and professional success [7,8].

Within this context, it has been argued it is paramount to address non-academic factors among higher education students so as to improve their engagement with their studies holistically [9]. In this sense Bowden et al. [10], conceptualised the four pillars of student engagement as behavioural engagement, affective engagement, social engagement and cognitive engagement. In addition, malleable factors of engagement included developing a growth mindset [11,12]. A sense of belonging for Baccalaureate nursing students was a pivotal concept which impacts student nurses and connectedness in clinical placements [13]. A students’ sense of belonging to the university had the most significant direct effect on their intention to stay or drop out [14], and extended to clinical placement collaboration [15]. In addition, self-efficacy, or the belief in one’s ability to succeed, enhanced persistence and effort.

Further, emotional intelligence, which involves managing one’s own emotions and understanding others’, is crucial for handling stress and social interactions [16]. For example, nursing students may experience emotionally challenging learning situations by the end of their undergraduate studies, and will need to further develop their emotional intelligence to meet the demands of managing social interactions and stressors of the role [17]. Lastly, effective learning strategies, such as goal-setting and self-assessment aid in knowledge acquisition and application. Addressing these factors provides a holistic approach to supporting student engagement and academic achievement [18,19,20].

Despite these efforts, during their first and second years at university, students often consider dropping out of their chosen course. While the reasons for this level of attrition is multifactorial, factors reported include exhaustion from the combination of intense study regimes to understand the theoretical elements of the programme, the demanding clinical placements, a lack of clinical supervision, reality shock of the clinical environment, and poor academic performance has also played a significant role amongst undergraduate nurse decision making [21,22,23]. Additionally, Pryjmachuk et al. [24] found age and educational qualifications predicted completion rates. Characteristics such as being less mature, being from a minority group, being male, and having lower qualifications were also associated with higher dropout rates. As such, large numbers of students choose to discontinue their studies before completing their degree which has implications for the passage of nurses into the future workforce [21].

### 1.1. A Potential Solution

To stem the attrition within nursing programmes globally, novel approaches are required to better support student academic success [18]. One such approach is the focus on incorporating self-care skills into the nursing curricular [25,26]. Mind–body practices can help to develop skills such as calmness and self-awareness, enabling individuals to become more connected to themselves and the people around them [27]. These mind–body practices can contribute to the development of non-cognitive factors, such as emotional regulation, resilience and stress management, which are essential for maintaining overall wellbeing and enhancing academic performance [1]. The foundation of a mind–body discipline suggests the more these techniques are practiced, an individual can develop a greater capacity to respond to stressful situations without reacting over emotionally or inappropriately to the present moment stimulus [28,29]. For example, non-cognitive factors of academic success are indicated to be improved through engagement with mind–body practices such as yoga and mindfulness [30,31,32,33].

Among these practices, yoga stands out as a set of techniques designed to bring calm, alert awareness to the mind and health and wellbeing to the body [34]. Yoga is officially recognised within the Nursing Interventions Classification (NIC) as a nursing intervention suitable for both healthy and ill individuals, along with its integration into nursing curricula [35]. Hatha yoga is a traditional form of yoga that emphasises physical poses and controlled breathing to prepare the body and mind for meditation. Hatha yoga is ideal for beginners due to its slower pace and focus on alignment and relaxation, which can generate benefits within a person at a physiological and psychological level. The benefits are found to result in better emotional states and reduce the levels of stress and anxiety in students, thereby improving their personal wellbeing [36]. In addition, the limited studies that explore the use of Hatha yoga to reduce stress in student nurses do show promise; however, most studies have been small samples which may limit their generalisability [37]. Despite these insights, there is a need for further research to better understand the impact yoga has on student success, specifically engagement (emotional, behavioural, cognitive, and social), procrastination, depression etc, and drop out among undergraduate nursing students.

### 1.2. Aim of Study

The aim of the study is to explore the impact of yoga on undergraduate nursing students’ engagement, depression, anxiety, stress, procrastination, sense of belonging, and intention to drop out, and to understand their lived experiences and perceived benefits of participating in yoga.

### 1.3. Research Questions

Within the context of the aim of the study, the research questions include:

1. What is the impact of yoga on undergraduate nursing students’ engagement, depression, anxiety, stress, procrastination, sense of belonging, and intention to drop out?
2. What are the lived experiences and perceived benefits of participating in yoga among undergraduate nursing students?

## 2. Materials and Methods

In line with the aims of the study, a convergent mixed-methods design was employed [38,39]. Specifically, a pre-test/post-test case series was utilised to measure outcomes before and after a Hatha yoga programme, providing a baseline for comparison and assessing the impact of the intervention on students. This approach was followed up with semi-structured interviews with students who volunteered to share their experiences participating in the programme. The benefit of the mixed-methods design is that it allows for triangulation of data and the results of the separate analyses are compared to determine if preliminary conclusions support each other [40]. The study has been guided by the STROBE guidelines to enhance the transparency of the research [41].

### 2.1. The Yoga Programme

The Hatha yoga programme was for eight weeks which aligns with established practices in psychosocial and behavioural nursing interventions, which commonly adopt this timeframe to ensure sufficient exposure and engagement while maintaining feasibility [42]. This approach, supported by the NIC, provides standardised guidance for intervention planning and duration, and is consistent with optimisation strategies for behavioural interventions outlined in the literature [35,42]. More specifically, the eight classes were pre-recorded and made available to the participants on a weekly basis. Typically, the class format lasted for 40–45 min of asana flows and 5–10 min of shavasana (restorative) at the end of the class.

The programme itself was developed for beginners and included foundational poses that encompassed standing, sitting, twists, forward bends and backbends [43]. Each class is designed to last approximately one hour, beginning with the physical postures and the sequences that would be included to cover standing poses, and concluding with 5–10 min of shavasana (deep relaxation) [43]. The sequence of classes gradually builds in complexity, allowing students to develop strength, flexibility, and confidence at a comfortable pace [43].

As part of the programme for beginners, there were weekly themes that progress from basic grounding and alignment to more dynamic flows and breath coordination. This then culminating in an integrated practice that encourages self-awareness, resilience and mindfulness with different activities which are built upon each week of the programme (Table 1). This format ensured participants not only learn the physical aspects of yoga but also experience its calming and restorative benefits. The course is ideal for those new to yoga or returning after a break, offering a supportive environment to explore movement, breath, and stillness [43].

The yoga instructor was a senior teacher trained in the Iyengar tradition within the broader Hatha yoga lineage. The Iyengar approach is primarily a form of modern postural yoga, offering a structured and systematic method for teaching asanas (postures) [44,45]. It is distinguished by its use of props, such as blankets, blocks, and straps, which enable students of all levels to practice safely and effectively in classical yoga postures. All participants were assumed to be beginners, and the programme was designed accordingly, with instruction tailored to a foundational level of understanding. Over the eight-week intervention, the sequencing of postures aimed to provide a comprehensive experience of asana practice, promoting overall wellbeing, stress reduction, and relaxation.

Overall, the programme adhered to the CLARIFY 2021 guidelines [46] for reporting yoga interventions. It included physical postures (asana) and restorative relaxation (shavasana), with no formal inclusion of pranayama or meditation. Classes were delivered asynchronously via a secure online platform, allowing participants to engage at their convenience. Props such as blocks, straps, and blankets were recommended, and modifications were demonstrated to accommodate beginner-level participants. While adherence was not formally tracked, participants were encouraged to complete one class per week. The intervention was designed to be accessible and supportive, fostering self-awareness and resilience through progressive sequencing and thematic focus [46].

### 2.2. Sample Participants

The study was conducted at an Australian university, which has metropolitan, regional, and rural campuses. Over a two-year period (2023–2024), all nursing students within the Baccalaureate nursing programme were invited to participate in the yoga programme and those who expressed interest (n = 59) were invited to participate in the study. To be eligible to participate in the study, the individual needed to be an undergraduate student at the participating university. Students were excluded if they were pregnant or suffered an injury, making it difficult to participate in the yoga (n = 0). Although not an exclusion criterion, each student was asked as part of the initial invitation and screening process, if they had practiced yoga previously (n = 0).

**Figure 1:**
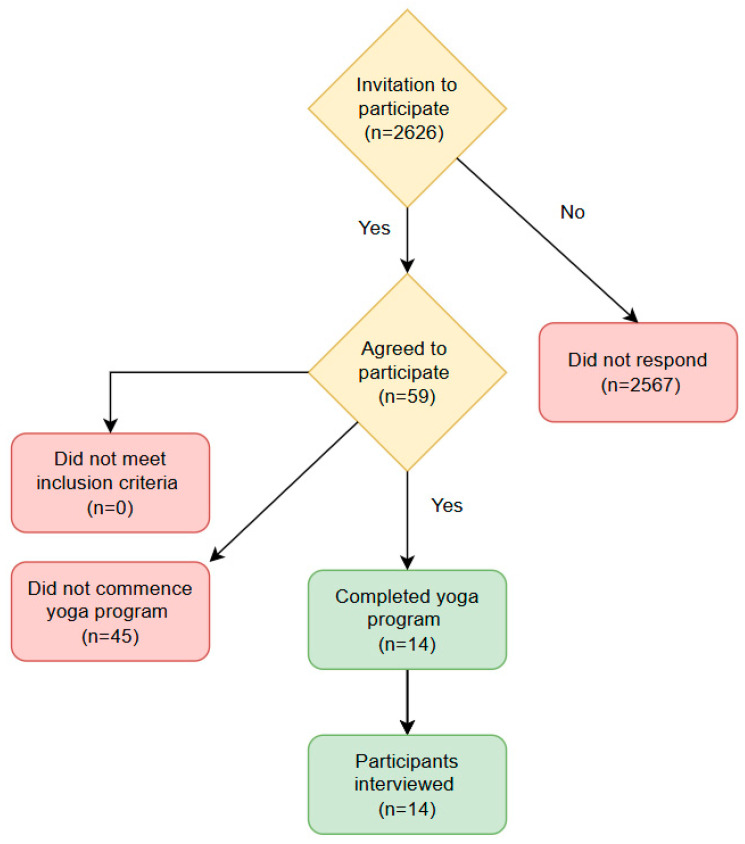
Participant recruitment, participation, dropout [19].

For the qualitative component of the study, sample size was determined based on the principle of saturation. Saturation was monitored throughout the data collection process and was considered achieved when participant responses began to repeat and no new themes or codes emerged. This supported the adequacy of the sample size and the robustness of the findings. While Guest et al. [47] suggest that code saturation can typically be reached after approximately nine interviews, and Hennick et al. [48] note that meaning saturation may require between 16 and 24 interviews, the research team (BM, DT, and BP) determined that 14 interviews were sufficient (Figure 1). This decision was based on the observation that additional interviews were not yielding any new insights, confirming that key themes were consistently represented across the dataset.

### 2.3. Data Collection

Data collection occurred between September 2023 and July 2024 coinciding with the various university teaching periods. Researchers provided university administration staff with an invitation letter to be emailed to all nursing students to ensure student anonymity. The invitation contained a web link with details about the yoga programme and interested students signed an informed consent form and completed the online questionnaire to ensure they met the inclusion criteria of the yoga programme. Follow-up recruitment emails were sent by the administration staff to the nursing students in weeks 1, 2, 4 and 6 after the initial invitation.

If students met inclusion criteria, they were invited to complete an anonymous online questionnaire prior to commencing the yoga programme and then again at the conclusion of the programme. All questionnaires were undertaken using Qualtrics software (Qualtrics©, Version May 2021). As part of the mixed-methods study students were invited to participate in a follow-up online interview regarding their experiences associated with the yoga programme and their nursing studies at the university. Semi-structured interviews were conducted within three weeks of each student completing their yoga programme and were achieved using interviews through video conferencing technology. All interviews were audio and video recorded and lasted between 30 and 60 min.

### 2.4. Data Collection Tools

Quantitative data were collected using a questionnaire that included demographic questions including gender, year of birth and country of origin. In addition, four standardised instruments were used to collect self-reported information from participants about their engagement at university, levels of depression, anxiety and stress, procrastination, sense of belonging and intention to drop-out. The instruments used included University Student Engagement Inventory (USEI); Depression, Anxiety, Stress Scale (DASS); Pure Procrastination Scale (PPS); General Belonginess Scale (GBS); and Intention To Drop Out Scale (IDO-3). Please see below for further details.

- The University Student Engagement Inventory (USEI) developed by Sinval et al. [49], which contains 15 items to measure three distinct constructs of academic engagement, encompassing behavioural, cognitive and emotional factors of student engagement. The USEI is scored on a five-point scale with categories ranging from 1-never to 5-always. The total score for USEI was fifteen-item University Student Engagement Inventory (USEI) where participants self-rate against fifteen items using a five-point Likert-type response (1-never to 5-always). Reliability coefficients in terms of the consistency of items are above 0.63 (ordinal omega values) and above 0.69 (ordinal values) for three dimensions [49].
- The Depression, Anxiety, Stress Scale (DASS) developed by Lovibond and Lovibond [50], which contains 21 items to measure depression, anxiety, and stress. The DASS was scored on a four-point scale with categories ranging from 0-never to 3-almost always. The total score for DASS was twenty-one items Depression, Anxiety, Stress where participants self-rate against twenty-one items using a four-point Likert-type response (0-never to 3-almost always). Alpha coefficients for 21-item DASS are as follows: Depression = 0.91, Anxiety = 0.84 and Stress [50].
- The Pure Procrastination Scale (PPS) developed by Steele [51], contains 12 items that measures procrastination. The PPS was scored on a five-point scale with categories ranging from 1-very seldom or not true of me to very often true or true of me. The total score for PPS was 12-item Pure Procrastination Stress (PPS) where participants self-rate against twelve items using a five-point scale with categories ranging from 1-very seldom or not true of me to true of me. These items were found to have high internal consistency Cronbach’s alpha a = 0.92 [52].
- The General Belonginess Scale (GBS) developed by Malone et al. [53], which contains 12 items to measure general belonginess. The GBS was scored on a seven-point scale with categories ranging from 1-strongly disagree to 7-strongly agree. The total score of GBS was 12-item General Belongingness Scale (GBS) where participants self-rate against twelve items using a seven-point Likert scale with categories. The items were found to have high internal consistency coefficient alpha = 0.92 and AIC = 0.49 (M = 70.0, SD = 10.9) [53].
- The Intention To Drop Out Scale (IDO-3) developed by Vallerand et al. [54], which contains 14 items to measure intention to drop out. The IDO-3 was scored on a four-point scale with categories ranging from rarely/not at all, sometimes, often, almost always. The total score for IDO-3 was 15-items where participants self-rate against fifteen items using a four-point Likert-type response (1-never to 5-always). These items were found to have high internal consistency, Cronbach’s alpha 0.79 [55].

Qualitative data were collected using semi-structured interviews. The interview guide was developed to align closely with the research aims and incorporated findings from within the literature [18]. Semi-structured questions were designed to elicit rich, narrative responses while allowing participants the flexibility to guide the conversation. The guide was refined through consultation with qualitative research literature, researcher team discussion, and pilot testing to ensure clarity, relevance, and sensitivity to participants’ contexts. Interview questions were specifically tailored to explore the impact of yoga on student engagement and wellbeing, with prompts designed to capture changes in emotional regulation, stress management, academic focus, and sense of belonging. The interview questions included key items such as ‘what has been your experience of participating in the yoga classes’; ‘what have you been able to integrate into your academic and personal life’; and ‘has there been any change to how you experience and feel the pressures of study?’ (Appendix A).

Participants who agreed to be contacted and responded to the invitation to be interviewed signed a consent form and provided additional verbal consent at the time of the interview. Microsoft Teams (Version: 2024.47.0-1.10-h452ab) was the audio-visual technology used during interviews with participant consent, primarily to increase accessibility students who were studying across the state, to also observe non-verbal responses, and to assist with transcription of the data. Interviews lasted between 20 and 60 min, with fieldnotes taken during and after each session.

Interviews were conducted by the principal researcher (BM), who was not a lecturer within the nursing programme, but teaching in another school and facility, so there was no previous or ongoing relationship with the nursing students which mitigated any potential or perceived power imbalance between the researcher and students. The principal researcher received support and guidance from the broader research team throughout the process. They practiced conducting interviews and received feedback from the other researchers after reviewing the recorded sessions to enhance technique and ensure consistency. The research team included four additional members (DT, BP, LR, and AD), all of whom have extensive post-doctoral experience in qualitative data collection and analysis, specifically associated with both health workforce and nursing student resilience and training [18,56,57,58]. Notably, one researcher (BM) has more than 20 years of experience in yoga and is an Iyengar trained yoga practitioner, while another author (AD) also has more than 10 years of experience of practicing and researching in the yoga space [59,60]. Both contribute valuable insight into the interpretation of participant responses and the contextual relevance of the yoga intervention.

Researcher reflexivity was considered throughout the data collection process to address any potential bias. The principal researcher (BM) maintained a reflexive journal, while also acknowledging her dual role as both yoga practitioner and researcher. This awareness informed the interview approach, with efforts made to minimise bias by using open-ended questioning and by engaging in regular discussions with the broader research team. Overall, this study adopted a hermeneutic phenomenological approach, which recognises the researcher’s interpretive role and encourages critical reflection rather than bracketing and a suspension of prior knowledge.

### 2.5. Data Analysis

Quantitative data were cleaned, checked, and analysed using Statistical Package for the Social Sciences (SPSS, Version 23.0). All responses for each of the five scales (USEI, DASS, PPS, GBS and IDO-3) were prepared in line with their specific requirements for assessment, whether overall or mean scores for sub scales or total scale scores [49,50,51,52,53,54]. Descriptive statistics were generated from the demographic data and were used to characterise responses to questionnaire items [59]. Inferential nonparametric statistics, such as Wilcoxon signed rank test, examined pre-and post-yoga group comparisons of paired samples, while Spearman’s rank correlation coefficient was used to examine correlation between scale items [61]. Correlation strength was defined as large (rho = 0.60–1.00), medium (rho = 0.40–0.60, and small (rho = 0.01–0.40). Significance was determined at two-tailed *p* ≤ 0.05 [61].

Qualitative data were transcribed into Microsoft Word (Version: 2024.30.01.04) to assist with data analysis, while Microsoft Excel (Version: 2408) was used to organise data extracts and support the initial coding process. Consistent with the phenomenological basis of the study, the qualitative data were analysed using reflexive thematic analysis, which was employed to identify key themes [62,63]. This approach involves identifying patterns of meaning across the dataset and developing themes that capture important aspects of participants’ experiences in relation to the research questions. A theme, in this context, represents a central organising concept that reflects shared meaning within the data [62,63].

Interview transcripts were read multiple times to identify key elements across the dataset. In line with Braun and Clarke [62,63], the researchers followed the six phases of thematic analysis: (1) familiarisation with the data, (2) generating initial codes, (3) searching for themes, (4) reviewing themes, (5) defining and naming themes, and (6) producing the report. The researcher’s role in knowledge production is central to thematic analysis [62,63]. The coding process involved ongoing reflexivity, including questioning and challenging assumptions made during interpretation. Through sustained analytic engagement, the researchers (BM and DT) independently identified patterns and themes. This systematic approach was chosen to minimise researcher bias and enhance the trustworthiness of the findings [62,63]. Reflexivity was a critical component of the research process, and Braun and Clarke’s method was selected for its flexibility and emphasis on researcher reflexivity. Participant data were labelled using pseudonyms and the year of interview (e.g., Sally, 2023; John, 2023; Mary, 2024).

To verify the accuracy and credibility of the data and interpretations, participants were invited to review the transcripts of their interviews and provide feedback on the accuracy of the recorded information. This process of member checking ensured that participants’ perspectives were accurately represented. These measures collectively contributed to the overall trustworthiness of the study [62,63].

### 2.6. Ethical Considerations

Ethical approval for this study was obtained from the Federation University Human Research Ethics Committee (Approval No. 2023/108) and the University of Southern Queensland Human Research Ethics Committee (Approval No. ETH2024-1102). All participants provided informed consent prior to their involvement in the study. Measures were taken to ensure participant confidentiality, and all data were de-identified to preserve anonymity throughout data collection, analysis, and reporting. After the study concluded, all identifiable information was securely stored in accordance with institutional data management policies to ensure ongoing confidentiality and anonymity.

## 3. Results

Overall, participants consisted of n = 59 students who completed the pre-yoga questionnaire, Participants included first-year students n = 22 (39%) second-year students, n = 25 (44%) and third-year students n = 10 (17%). Participants included n = 40 (68%) full-time and n = 19 (32%) part-time students. Most students were female n = 56, (95%) and were either born in Australia n = 38 (64%) or overseas n = 21 (36%). It must be noted only 14 (22%) of students completed the yoga programme and the final questionnaire, while the remaining students were lost to follow up. Further it must be noted among those who completed the yoga programme and post-programme questionnaire, there were no specific groups that were underrepresented, except for males (Table 2).

### 3.1. Correlation Between Student Engagement, Belongingness, Depression, Procrastination and Intention to Dropout

When examining participant responses to key questionnaire items prior to participating in the yoga programme, it was found that several key items correlated. Within this context it was found that depression strongly correlated negatively with behavioural engagement (ϼ = −0.439, *p* = 0.001) and emotional engagement (ϼ = −0.529, *p* = 0.001) among all students. In addition, general belonginess was also found to have a strong negative correlation with depression (ϼ = −0.733, *p* = 0.001), along with anxiety (ϼ = −0.701, *p* = 0.001) and stress (ϼ = −0.638, *p* = 0.001) among students. Further, among the many items where correlation was present, it was further noted that there was a moderate positive correlation between procrastination and intention to drop out (ϼ = 0.341, *p* = 0.001) (Appendix B).

### 3.2. Depression, Anxiety, and Stress Among Students Before and After the Yoga Programme

The Wilcoxon signed rank test was employed to examine the differences in mean rank scores for depression, anxiety and stress along with other key items before and after the yoga intervention. For depression, anxiety and stress, the rank scores significantly decreased among participants when comparing before and after participation in the yoga programme. However, it must be noted that no other measures demonstrated significant differences between pre- and post-yoga programme testing scores. This suggests yoga may have had a positive impact reducing depression, anxiety, and stress, with a positive impact on other scale items (Table 3).

When discussing the impact of yoga, they expressed that due to the years of dealing with negativity in their life participation in yoga has assisted them to keep going:

*Prior to doing yoga I would be tired and exhausted both mentally and physically. After yoga I could accomplish and do things and the body could keep going, rather than flop down and just do nothing.* (Participant 7)

In addition, yoga really provided them with a sense of drive and achievement. In addition to assisting with the negative aspects of their lives which each of them was struggling with, in some cases, years, there were other benefits that impacted their mental health.

*I need to take a lot more time for myself during the day which means to relax and calm down… I forget to put me first sometimes. Since doing the 8-weeks of yoga, it has become a lot more important to make sure I am healthy and well in order to look after other people*. (Participant 1)

The experiences among the students highlight the profound impact yoga has had on their lives, providing them with a renewed sense of purpose and wellbeing. Through consistent practice, they have found the strength to overcome long-standing negativity and improve their mental health. Yoga has not only helped manage their struggles but also empowered them to prioritise their own health and wellbeing.

### 3.3. Engagement Among Students Before and After the Yoga Programme

The differences in mean rank scores for behavioural and emotional engagement before and after a yoga intervention were examined. Among those who participated in yoga were more likely to have significant higher levels of behavioural and emotional engagement, than those who did not commence the programme. The results suggest yoga may have a positive impact on increasing both behavioural and emotional engagement among participants who completed the programme; however, no impact on cognitive engagement was shown (Table 4).

The 8-week programme intrinsically required a commitment to the research by participating in the yoga classes. For participants who completed the programme this was evidence of the effort required as participants were already heavily committed with work, family and study priorities. Some participants described the profound effort to stay engaged and found strategies such as getting their children to join them viewing the video.

*When I started this yoga thing it was amazing. An hour to focus on something I enjoyed. Once we got into the semester it became harder so I would get my son to join me with some of the yoga and he loved it too*. (Participant 4)

In addition, participants further stated the skills they had learned from the yoga, and included moving through the yoga poses, with focus and concentration.

*Learning how to proactively engage in the work, while also existing. It is a complete change of mindset. I am still working on it, but it has definitely started… Everything is repetition right to learn a new skill and change a habit which has been with me a lifetime.* (Participant 4)

This finding aligns with quantitative data where participants who persevered through the programme, despite juggling multiple responsibilities, experienced higher engagement levels. The commitment to yoga not only fostered behavioural and emotional engagement but also facilitated a shift in mindset, demonstrating how integrating such practices can enhance learning and personal growth.

### 3.4. Comparison Among Australian and Overseas Students Before and After the Yoga Programme

Further analysis was undertaken to examine the differences in mean rank scores for depression, anxiety, and stress along with engagement and other items before and after the yoga intervention by comparing students by age groups, study modes (full-time and part-time), and place of birth. While sample sizes across age groups were insufficient and differences by study mode were not statistically significant, place of birth showed that mean rank scores for stress significantly decreased among participants born in Australia. This was not observed among participants born overseas, and no other scale items showed significant differences. When examining students born overseas, the mean rank scores for behavioural and cognitive engagement significantly increased, while there was no significant difference with other scale items. These results suggest that yoga may have a more pronounced impact on reducing anxiety among participants born in Australia, while yoga has a more positive impact on behavioural and cognitive engagement among those born overseas (Table 5).

When discussing the effects of yoga on anxiety an Australian participant who had previously been living in a very small world of her own experienced reduced feelings of being isolated when she reached out to join the yoga group study:

*Prior to yoga I literally wasn’t living. I lived in my own little world and then sort some professional help. The psychiatrist recommended yoga. My mind was going 24/7. I took that step. I did not have the confidence to do face to face yoga as I don’t feel confident*. (Participant 7)

In relation to differences between Australian and Overseas students for behavioural and cognitive engagement, overseas participants experienced greater engagement following participation in the yoga programme. For overseas students who were working in aged care the body was challenged as moving bedridden patients, which was described as hard work, as bodies were heavy to move. Participants were searching for ways to release their bodies which required engaging with the programme for 8-weeks to learn how to stretch and relax the body. This was made clear by one international student who stated:

*The key for me was to release the muscles of my body as I work in aged care and many people are confined to their beds. There is a lot of pushing and pulling required by the nurses to assist patients move to a new position. In the beginning I found yoga was hard on my body as I was not used to stretching and I thought ‘no, I cannot do this’. However, after the yoga, which I did in the morning, I felt mentally fresh for the day. I have not done any stretching for a long time and my shoulder [was] getting worse I found the yoga helped with reducing the pain*. (Participant 13)

When discussing the importance of stretching the body this participant further explained that she had been moving people around for a very long time in her job and was at the stage when the body was getting very stiff and tired.

*If I do yoga in the morning I can do those other things. When you feel tired your body is saying no. It is a ‘mind-freshing’ experience, also with the breathing and stretching the body. My son helped me as well, he says this is not the angle they are showing, and he assisted me to get into the poses*. (Participants 13)

This finding aligns with quantitative data indicating that overseas participants experienced greater behavioural and cognitive engagement which could be contributed to the importance of learning ways to work with their body to overcome the demands placed upon their bodies in their work environment. Yoga has significantly improved students’ mental health and wellbeing, helping them overcome long-standing negativity and prioritise self-care. Differences were observed between Australian and overseas participants, who reported reduced anxiety and increased engagement, particularly those in physically demanding jobs, and who found yoga essential for managing their work-related stress and physical strain.

## 4. Discussion

The current study examined the efficacy of an 8-week yoga intervention among undergraduate nursing students’ engagement, depression, anxiety, and stress levels. The aim of the study was to seek to understand the role of yoga regarding key factors linked to student success and academic performance. As such, a reduction in depression, anxiety, and stress was demonstrated to occur among those nursing students who participated in the eight sessions of hatha yoga exercise. In addition, behavioural and emotional engagement significantly increased among those nursing students who participated in the yoga programme. These findings resonate with current research where a growing interest in yoga has been found to be beneficial for cultivating a more sustained holistic wellbeing and maintaining current health for nursing students [1,37,64].

Overall, the study found significant improvements in self-reported measures of depression, anxiety, and stress levels among nursing students following the 8-week Hatha yoga intervention. However, data on the neurobiological and behavioural mechanisms underlying these effects remain scarce [65]. Nevertheless, these findings suggest that yoga can be an effective intervention for reducing depression, anxiety, and stress among nursing students. This aligns with previous studies that have shown yoga’s benefits for mental health through mechanisms such as mindfulness, self-compassion, and improved self-regulation [26,66,67,68]. Further, the students reported that yoga helped them manage long-standing negativity and provided a sense of accomplishment, which aligns with the observed reductions in depression, anxiety, and stress. For example, participants mentioned feeling more capable and driven after yoga sessions, while others highlighted the importance of self-care and prioritising personal wellbeing. These insights highlight the positive psychological impact of yoga. As such, the results imply that integrating yoga programmes into nursing curricula may enhance students’ mental health and academic performance by promoting self-regulation, mindfulness, and stress reduction.

In addition, the study found significant improvements in both behavioural and emotional engagement among nursing students after completing a Hatha yoga programme. This aligns with previous research indicating that behavioural and cognitive engagement are crucial for academic success [69]. These findings suggest that incorporating Hatha yoga into nursing education can enhance students’ engagement and academic performance [70]. This supports Farahani et al. [71], who found that ‘Super Brain Yoga’ improved concentration, memory, and academic development in nursing students.

The 8-week programme required a significant commitment from participants, who were already balancing work, family, and study priorities. Some participants described the effort to stay engaged, such as involving their children in the yoga sessions. Additionally, participants highlighted that the skills learned from yoga, such as moving through poses, required focus and concentration. These insights demonstrate participants who persevered through the programme experienced higher engagement levels. The commitment to yoga not only fostered behavioural and emotional engagement but also facilitated a shift in mindset, demonstrating how integrating such practices can enhance learning and personal growth. As such, the results imply that integrating yoga programmes into nursing curricula could foster essential skills such as academic mastery, critical thinking, and emotional maturity. This holistic approach may better prepare nursing students for clinical education and professional practice. Future research should explore the long-term effects of such interventions and their potential to enhance overall academic success.

However, it is important to acknowledge that the high dropout rate observed in this study may reflect broader challenges in nursing education related to self-commitment. While yoga shows promise as a wellbeing intervention, its effectiveness may be limited by students’ capacity to engage consistently, particularly during periods of academic intensity. This is supported by Kinchen et al. [37] who found that students self-selected into a yoga programme and that engagement varied, with no significant improvements in stress or quality of life among the broader cohort [37]. This self-selection bias suggests that those who completed the programme may have already possessed higher levels of motivation and self-regulation, potentially influencing the positive outcomes observed [37]. Therefore, while yoga may be a suitable technique for some, its scalability and accessibility require further exploration. Sudhan and Parveen [72] demonstrated that online yoga programmes can be effective in reducing stress and improving emotional regulation, suggesting that digital delivery may help overcome barriers to participation. Future research is needed to explore ways to embed such practices into the curriculum to reduce barriers to participation and better support students who may struggle with self-commitment.

Lastly, it was also found that yoga significantly reduced anxiety among Australian-born nursing students, while it enhances behavioural and cognitive engagement among overseas-born students. For example, an Australian participant who had previously felt isolated reported reduced feelings of isolation after joining the yoga programme. This may be due to cultural differences impacting how individuals perceive or engage with the yoga, while also be due to differences in work environments, social supports, or stress levels among the different cohorts [73]. Further, overseas-born students may encounter additional challenges related to social integration and support, thus by participating in yoga may help them feel more connected and supported, thereby increasing their engagement [74]. As such, overseas participants stated they experienced greater levels of engagement following participation in the yoga programme, and for overseas students working in aged care, the physical demands of their job made yoga particularly beneficial. These results align with previous research highlighting yoga’s benefits for mental health and engagement [75,76]. This indicates that incorporating yoga into nursing education programmes may provide tailored benefits, improving mental health and engagement based on students’ cultural backgrounds. However, future research may seek to explore the long-term effects of yoga interventions and their potential to improve overall wellbeing and academic success.

### 4.1. Limitations

There are several limitations within this research that must be recognised, where a key limitation was the timing of the research. Although COVID-19 was officially declared ‘over’ in Australia on 20 October 2023, there was a backlog of student Clinical or Professional Experience Placeement (PEP) due to the pandemic, and this impacted future placements [77]. Initially, 59 students expressed interest in participating in the yoga programme. However, to expedite the PEP backlog, the teaching period where the study was conducted had been condensed from twelve weeks to six weeks. This created an academic overload, preventing students from continuing their commitment to their studies and the yoga programme.

A key limitation of this study was the small sample size, despite multiple rounds of recruitment and follow-up with students who initially expressed interest. Sustained engagement proved challenging, particularly during a large change in a study programme that led to periods of academic intensity. Notably, the high attrition rate must be considered beyond its logistical implications. While it highlights the difficulty of implementing wellbeing interventions during demanding academic periods, it may also reflect a self-selection bias. Further, future studies with larger sample sizes should explore characteristics such as age, place of birth, and study mode more robustly, as these may provide greater insights into the impact of yoga among nursing students.

Students who completed the programme were likely those already motivated to enhance their wellbeing, potentially contributing to the positive outcomes observed. This may impact the generalisability of the findings, as the intervention may be less effective or appealing to students with lower initial motivation. Future research should explore strategies to enhance engagement and retention, particularly among students who may benefit most from such interventions but are less likely to persist. Lastly, the findings of the study are specific to the context of the university where the study was conducted and may not be generalisable to other universities or nursing programmes with different structures and student demographics.

### 4.2. Future Research

This study builds on previous research demonstrating the benefits of yoga for managing stress, anxiety, and depression. However, future research should further investigate these differential impacts and consider integrating yoga and other contemplative practices into the nursing curriculum to enhance students’ overall wellbeing and professional development. The favourable results highlight the positive impact of introducing a foundational yoga unit into the first-year nursing curriculum.

## 5. Conclusions

In this study, nursing students demonstrated a reduction in depression, anxiety and stress and an increase in behavioural and cognitive engagement after completing an 8-week yoga programme. These results suggest that adopting a regular programme of yoga in the undergraduate course is beneficial for students’ self-care and long-term psychological wellbeing. The information gathered from our research may assist in designing curricular opportunities within the Bachelor of Nursing programme to support students to develop positive coping mechanisms or skillsets to navigate their academic and life stressors; however, this information must be used cautiously as the findings may not be generalisable to other nursing student populations.

## Figures and Tables

**Table 1:** Outline of the yoga programme.

Curriculum	Focus Area	Key Themes
1	Mountain pose	Stability and balance.
2	Recovery poses	Ardha Uttanasana, Adho muka svanasana, Prasarita Padottonasana, Viparita Karani.
3	Trikonasana (triangle pose)	Learning to bend the trunk sideways and turn the trunk and neck. Learning alignment of the legs and arms.
4	Virabhadrasana 11	Learning to bend the leg to a square against the stretched leg without allowing the leg to learn towards the bent leg. Learning to coordinate these opposite actions.
5	Parsvakonasana	Learning to keep the structure of the thoracic chest broad and with a bent front leg.
6	Ardha Chandrasana	Learning to shift the weight of the body on the right hand and the right leg. Learn to lift the left leg and stretch the right leg simultaneously.
7	Parsvotanasana	Learning to bend forward giving a forward extension to the spinal muscles with a concave back.
8	Prasarita Padottanasana	Learning to spread the legs apart without allowing the feet to slide and slip off giving a forward extension to the spinal muscles.
9	Uttanasana	Learning to lengthen the sides of the trunk with a firm grip on the elbow.
10	Buddha Konasana	Learning to open the groins and loosen the hips joints.
11	Upavistha Konasana	Learning to lift and concave the spine.
12	Svastikasana	Learning to cross and uncross the legs changing their position alternately.
13	Arm variations	Learning to open the armpit chest and move the shoulder-blades in.
14	Janu Sirsasana	Learning to extend the lumber trunk by making the dorsal spine concave.
15	Savasana	Learning to lie down rest the body which includes integrating the lesson in stillness.

**Table 2:** Characteristics of undergraduate nursing students.

Demographics (n = 59)	Number (n)	Percentage (%)	Number (n)	Percentage (%)
	Pre-Yoga Programme		Post-Yoga Programme	
**Gender**				
Female	56	95%	14	100%
Male	3	5%	0	0%
**Age group**				
Under 29 years	27	46%	6	42%
30–39 years	15	25%	2	14%
40–49 years	7	12%	2	14%
50 years and older	10	17%	4	28%
**Birthplace**				
Australia	38	64%	11	79%
Overseas	21	36%	3	21%
**Study year**				
Year 1	22	37%	3	21%
Year 2	25	43%	7	50%
Year 3	10	17%	4	29%
Missing	2	3%	0	0%
**Study mode**				
Full-time	40	68%	9	64%
Part-time	19	32%	5	36%

**Table 3:** Differences in mean rank scores of depression, anxiety and stress before and after yoga.

	Test Statistic (Wilcoxon)	Pre-Yoga Mean Rank (n)	Post-Yoga Median Scores (n)	*p*-Value
Depression	181.500	30.29 (n = 43)	20.96 (n = 13)	0.050 *
Anxiety	143.500	30.83 (n = 44)	18.46 (n = 12)	0.016 *
Stress	179.500	30.93 (n = 44)	20.81 (n = 12)	0.042 *
Rejection exclusion	227.500	26.32 (n = 42)	29.54 (n = 12)	0.609
Acceptance	203.500	25.85 (n = 42)	31.54 (n = 12)	0.312
Intention to drop out	202.000	26.44 (n = 42)	23.33 (n = 12)	0.469
Procrastination	157.000	24.90 (n = 36)	20.27 (n = 11)	0.302

* *p* ≤ 0.05.

**Table 4:** Differences in behavioural and emotional engagement before and after yoga.

	Test Statistic (Wilcoxon)	Pre-Yoga Median Scores (n)	Post-Yoga Median Scores (n)	*p*-Value
Behavioural Engagement	178.500	28.22 (n = 48)	41.27 (n = 12)	0.018 *
Emotional Engagement	179.500	27.82 (n = 47)	40.19 (n = 12)	0.023 *
Cognitive Engagement	228.000	28.85 (n = 47)	36.46 (n = 12)	0.161

* *p* ≤ 0.05.

**Table 5:** Differences between Australian and Overseas students before and after yoga.

	Place of Birth	Test Statistic (Wilcoxon)	Pre-Yoga Mean Rank Score	Post-Yoga Mean Rank Score	*p*-Value
Behavioural Engagement	Australia	99.500	18.43	24.55	0.139
	Overseas	195.000	10.26	19.33	0.024 *
Emotional Engagement	Australia	93.000	17.82	24.20	0.116
	Overseas	201.000	10.58	17.33	0.091
Cognitive Engagement	Australia	118.500	18.73	21.65	0.469
	Overseas	196.500	10.34	18.83	0.034 *
Depression	Australia	92.500	20.57	14.75	0.143
	Overseas	18.000	10.75	6.00	0.176
Anxiety	Australia	83.000	20.93	13.80	0.073
	Overseas	9.500	10.62	4.75	0.156
Stress	Australia	69.500	21.43	12.45	0.024 *
	Overseas	26.500	10.79	8.83	0.592
Rejection Exclusion	Australia	113.000	18.63	20.00	0.731
	Overseas	131.000	8.87	10.00	0.765
Acceptance	Australia	125.000	18.19	21.20	0.451
	Overseas	133.000	8.73	11.00	0.549
Intention to drop out	Australia	105.000	19.46	16.00	0.371
	Overseas	16.000	8.00	8.00	1.000
Procrastination	Australia	90.500	20.02	14.55	0.162
	Overseas	57.500	5.75	8.50	0.427

* *p* ≤ 0.05.

## Data Availability

The data presented in this study are available on request from the corresponding author as the data are not publicly available due to privacy or ethical restrictions.

## Public Involvement Statement

No public involvement in any aspect of this research.

## Guidelines and Standards Statement

This manuscript was drafted against the Strengthening The Reporting of Observational Studies in Epidemiology (STROBE) guidelines for quantitative research.

## Appendix A. Semi-Structured Interview Guide

1. You have been attending a yoga class for eight sessions. Tell me what your experience of has been like in the class.
2. What have you learned about yourself from your experience of attending the yoga classes so far?
3. What aspects of the class have you enjoyed or found challenging.
4. Were you able to follow the instructions from the yoga teacher?
5. What insights did you gain from the yoga classes?
6. In what ways has participating in the yoga programme impacted or not impacted your studies, in terms of feeling happier, feeling like you belong as a university student, or even thinking about dropping out?

## Appendix B

**Table A1:** Spearman’s rank correlation between students’ pre-yoga intervention.

		Behavioural Engagement	Emotional Engagement	Cognitive Engagement	USEI	Depression	Anxiety	Stress	Rejection Exclusion	Acceptance	GBS (Total)	Intention to Drop Out
Emotional Engagement	rho	0.686 **										
	Sig	0.001										
	n	47										
Cognitive Engagement	rho	0.630 **	0.601 **									
	Sig	0.001	0.001									
	n	47	47									
USEI (Total)	rho	0.877 **	0.874 **	0.863 **								
	Sig	0.001	0.001	0.001								
	n	47	47	47								
Depression	rho	−0.439 *	−0.529 **	−0.383	−0.507 **							
	Sig	0.003	0.001	0.011	0.001							
	n	43	43	43	43							
Anxiety	rho	−0.445 *	−0.452 *	−0.186	−0.405 *	0.790 **						
	Sig	0.002	0.002	0.226	0.006	0.001						
	n	44	44	44	44	43						
Stress	rho	−0.338 *	−0.404 *	−0.232	−0.367 *	0.782 **	0.832 **					
	Sig	0.025	0.006	0.129	0.014	0.001	0.001					
	n	44	44	44	44	43	44 **					
Rejection Exclusion	rho	0.398 *	0.462 *	0.388 *	0.473 *	−0.670 **	−0.597 **	−0.564 **				
	Sig	0.009	0.002	0.012	0.002	0.001	0.001	0.001				
	n	42	41	41	41	40	41	41				
Acceptance	rho	0.458 *	0.494 **	0.274	0.462 *	−0.733 **	−0.701 **	−0.638 **	0.914 **			
	Sig	0.002	0.001	0.083	0.002	0.001	0.001	0.001	0.001			
	n	42	41	41	41	40	41	41	42			
GBS (Total)	rho	0.438 *	0.489 * *	0.337 *	0.478 *	−0.719 **	−0.663 **	−0.614 **	0.978 **	0.979 **		
	Sig	0.004	0.001	0.031	0.002	0.001	0.001	0.001	0.001	0.001		
	n	42	41	41	41	40	41	41	42	42		
Intention to drop out	rho	−0.642 **	−0.744 **	−0.397 *	−0.673 **	0.484	0.500 **	0.370 *	−0.456 *	−0.524 **	−0.500 **	
	Sig	0.001	0.001	0.012	0.001	0.002	0.001	0.020	0.004	0.001	0.001	
	n	39	39	39	39	38	39	39	39	39	39	
Procrastination	rho	−0.560 **	−0.480	−0.335 *	−0.543 **	0.575 **	0.596 **	0.581 **	−0.506 *	−0.575 **	−0.553 **	0.341 *
	Sig	0.001	0.003	0.046	0.001	0.001	0.001	0.001	0.002	0.001	0.001	0.042
	n	36	36	36	36	36	36	36	36	36	36	36

* *p* ≤ 0.05; ** *p* ≤ 0.001.
